# Number to me, space to you: Joint representation of spatial-numerical associations

**DOI:** 10.3758/s13423-021-02013-9

**Published:** 2021-11-23

**Authors:** Stefania D’Ascenzo, Martin H. Fischer, Samuel Shaki, Luisa Lugli

**Affiliations:** 1 Department of Philosophy and Communication, University of Bologna, Bologna, Italy; 2 Department of Psychology, University of Potsdam, Potsdam, Germany; 3 Department of Behavioral Sciences, Ariel University, Ariel, Israel

**Keywords:** Social co-representation, Conceptual congruency effect, Numerical cognition, SNARC effect

## Abstract

Recent work has shown that number concepts activate both spatial and magnitude representations. According to the social co-representation literature which has shown that participants typically represent task components assigned to others together with their own, we asked whether explicit magnitude meaning and explicit spatial coding must be present in a single mind, or can be distributed across two minds, to generate a spatial-numerical congruency effect. In a shared go/no-go task that eliminated peripheral spatial codes, we assigned explicit magnitude processing to participants and spatial processing to either human or non-human co-agents. The spatial-numerical congruency effect emerged only with human co-agents. We demonstrate an inter-personal level of conceptual congruency between space and number that arises from a shared conceptual representation not contaminated by peripheral spatial codes. Theoretical implications of this finding for numerical cognition are discussed.

## Introduction

The ubiquitous link between number magnitudes and space has been intensively investigated. Typically, participants classify centrally presented digits as odd or even (or as smaller or larger than 5) with lateralized response keys, and classify small numbers (i.e., 1 and 2) faster with left-side responses and larger numbers (i.e., 8 and 9) faster with right-side responses (Dehaene et al., 1993). This performance signature of spatial-numerical associations (SNAs) implies that number knowledge is inherently spatially represented on a “mental number line” (Fischer & Shaki, 2014; Toomarian & Hubbard, 2018).

Is it possible that SNAs merely reflect extraneous spatial task demands and are not an inherent part of the number representations themselves? Indeed, most previous studies included either spatially distributed responses (as the seminal study by Dehaene et al., 1993 and many replications) or lateralized additional stimuli (e.g., detection probes for attention measurement in Fischer et al., 2003). Recently, Fischer and Shaki (2016, 2017) addressed this methodological concern and demonstrated SNAs with non-spatial stimuli and non-spatial responses. They used a modified go/no-go task with central stimuli and a single central response key. Specifically, stimuli from two categories (numbers and spatially oriented objects) were randomly presented and participants responded according to a previously agreed rule that combined a magnitude-related instruction for numbers with a direction-related instruction for objects (e.g., “respond for numbers < 5 or objects facing left”). SNAs were measured under four different conjunction rules (</left, </right, >/left, and >/right), always for centrally presented numbers and with central responses. Importantly, the spatial feature of objects was irrelevant and absent when responses were recorded, since participants replied only to number magnitude. The congruency benefit for conjunctions smaller/left and larger/right established a purely conceptual link between numbers and space.

Following these demonstrations of purely conceptual SNAs, two recent studies further investigated whether space is inherent in number meaning or artifactually activated due to spatial task requirements. Note that, in the above go/no-go procedure, spatial-directional processing is absent during number processing, but the number-related part of the response rule requires explicit magnitude processing. Shaki and Fischer (2018) compared SNAs in the go/no-go procedure for explicit and implicit magnitude processing by measuring performance with magnitude-related and parity-related rules for numbers, respectively. SNAs appeared again when magnitude processing was explicit but were absent when participants evaluated parity. Thus, when number meaning is implicit and the task is non-spatial, there are no SNAs. We must explicitly activate *at least one* of the two components of that association – either the spatial-directional or the magnitude component – to associate numbers with space.

Pinto, Pellegrino, Marson, et al. (2019a) extended this insight based on their own independent hypotheses (previously advanced in Aiello et al., 2012; Fattorini et al., 2015; and Pinto et al., 2018; see also Pinto, Pellegrino, Lasaponara, et al., 2019b; Pinto et al., 2021), and clarified that *both* space and magnitude must be used in conjunction to trigger SNAs. Activating a single task component alone (e.g., magnitude, by instructing participants to “respond for numbers < 5, or for all arrows”) was not sufficient to generate SNAs. Instead, both spatial-directional and magnitude components were required in conjunction (e.g., “respond for numbers < 5, or for *left* arrows”) to evoke SNA. Interestingly, the conceptual association is often asymmetrical to one side (Pinto, Pellegrino, Marson, et al., 2019a: only for left space–small numbers; Li & Pan, 2020: only for upper space–powerful words).

Here we further elaborate on representational constraints on SNAs, asking whether the two necessary components, explicit magnitude meaning and explicit spatial coding, must be present in a single mind, or whether they can be distributed across two minds. This important question arises from an influential theoretical framework highlighting the importance of social co-representation. Participants typically represent task components assigned to others together with their own task responsibilities, even when inter-personal coordination is not required (e.g., Sebanz et al., 2003). Evidence for shared task representations emerged in various settings (e.g., Simon task, Sebanz et al., 2003; Flanker task, Dolk, Hommel, Prinz, & Liepelt, 2014a; Stroop task, Yamaguchi, Clarke, & Egan, 2018a; and task switching, Yamaguchi et al., 2017a). In addition, many studies focused on what is shared in terms of task, goals, and actions (e.g., Yamaguchi, Wall, & Hommel, 2018b, 2019a).

Sebanz et al. (2003) showed first (but see also Sebanz et al., 2005) how participants incorporate others’ instructions, thereby affecting their own performance. Consider first the standard Simon task (Simon & Rudell, 1967), where participants use left/right keys to classify non-spatial stimulus attributes (e.g., red/blue dots) that randomly appear on the left/right side. Although stimulus location is task-irrelevant, responses are faster when they spatially correspond to the stimulus position (i.e., Simon effect). In a go/no-go version when individual participants respond only to one of two stimuli (e.g., “left key for red dots”) and ignore the other stimulus completely, the Simon effect disappears (Hommel, 1996). However, when each of two participants responds to one half of all stimuli (e.g., participant_1 follows the rule “left key for red dots” and participant_2 follows the rule “right key for blue dots”), a “social Simon effect” returns (Sebanz et al., 2003). It seems to emerge from introducing feature overlap in one’s own and the co-participants’ response representations. Thus, adding a second participant created a shared spatial framework (for confederate effects, see also Dudarev & Hassin, 2016; Maehara et al., 2019). Several studies were conducted to understand the nature of shared spatial representation underlying the social Simon effect (cf. Dolk, Hommel, Colzato, et al., 2014b).

Extending this rationale to number-related paradigms, Atmaca et al. (2008); see also Zhang et al., 2012), in their joint condition, assigned each participant one response key (left/right) and one parity (odd/even). The authors found that the spatial attribute of number representations can be co-represented: Not only my own hands are left/right; even with single responses, each participant locates himself left/right of another participant. Similarly, Towse et al. (2016) and Hartman et al. (2019) reported social effects during random number generation and showed that cognitive access to numbers depended on spatially coding another participant.

The present study extended this recent work by conducting a social version of Fischer and Shaki’s (2016)go/no-go procedure to evaluate the possible co-representation of spatial and magnitude codes in the generation of the SNA. The task requires each participant to be in charge of only one code (numerical or spatial) by responding to either number magnitudes or arrow directions. The response rules were:

Participant_A: “respond for numbers < 5”; Participant_B: “respond for left arrows”;

Participant_A: “respond for numbers > 5”; Participant_B: “respond for right arrows”;

Participant_A: “respond for numbers > 5”; Participant_B: “respond for left arrows”;

Participant_A: “respond for numbers < 5”; Participant_B: “respond for right arrows”;

where the first two rules are congruent and the last two incongruent with typical SNAs expressed through the mental number line metaphor. Importantly, for Participant_A instructions render the task spatially neutral with regard to all congruency considerations because he/she only responds to a single central stimulus type on a single central button while facing another person frontally. This generates a situation in which all congruency effects must be attributed to a shared conceptual framework, thereby revealing joint conceptual congruency across two minds.

We predicted that Participant_A would co-represent the other person’s response rule and respond faster in congruent conditions so that SNAs would occur even when the two codes were shared between minds (i.e., human co-agent condition). Since we were not interested in the processing of arrows, we used a confederate as Participant_B.

Based on previous studies that employed the standard Simon joint action paradigm using either a real co-actor or a computer program (e.g., Tsai et al., 2008), we also investigated the robustness of the hypothesized co-representation: In a second condition (i.e., non-human co-agent) Participant_A was paired with a computer. If the co-representation is restricted to conspecifics, the conceptual congruency effect should only occur when interacting with a human partner. It is important to note that many tasks in the social cognition literature (e.g., Yamaguchi et al., 2017b; see also Yamaguchi, Welsh, et al., 2019b) involve task-goal sharing (getting to a destination: driver leads, co-driver gives directions) or action-goal sharing (boating: all those who row must coordinate their actions). Even in the above reviewed social Simon (i.e., Sebanz et al., 2003) and social SNARC effects (i.e., Atmaca et al., 2008), the participants share a spatial framework. However, the only shared component in our study is the extent to which the experimental situation is conceptualized as spatially numerically congruent or not. Manipulating the type of co-agent (human vs. computer) is not relevant for the main goal of discovering shared conceptual congruency; instead, it addresses the independent question of possible limitations of such joint conceptual SNAs.

## Materials and methods

### Participants

Based on Pinto, Pellegrino, Marson, et al. (2019a) and on effect sizes obtained by Shaki and Fischer (2018, Experiment 2), the sample size needed to obtain a power of 0.89 with alpha set to 0.05 (two-sided) was 24 (Cohen's d=0.68) per condition. Forty-eight students of University of Bologna, 24 for the human co-agent condition (13 females, right-handed, M_age_ = 20.6 years, SD_age_ = 1.8) and 24 for the non-human co-agent condition (20 females, one left-handed, M_age_ = 19, SD_age_ = 0.7) were tested. All reported normal or corrected-to-normal vision and were naïve regarding the experiment’s purpose. The study was conducted according to The Code of Ethics of the World Medical Association (Declaration of Helsinki). The study was approved by the Bioethical Committee of the University of Bologna, and all participants provided written informed consent.

### Stimuli, apparatus, and design

Stimuli were four digits (1, 2, 8, 9; size 1.5 × 1.8 cm) and two arrows (pointing left or right; size 2.5 × 1.8 cm) presented in black on white background on a 15-in. laptop monitor with 1366 × 768 pixel resolution. The space bar of a QWERTY keyboard recorded responses. Two identical laptops (Notebook Toshiba Satellite PRO C660-1ZF) were arranged at opposite ends of a table. The distance between screens was 70 cm and that between participants (face to face) approximately 180 cm. E-Prime®software (Schneider et al., 2002) controlled stimulus presentation and response collection on the participant’s laptop while no software ran on the confederate’s laptop.

**Fig. 1:**
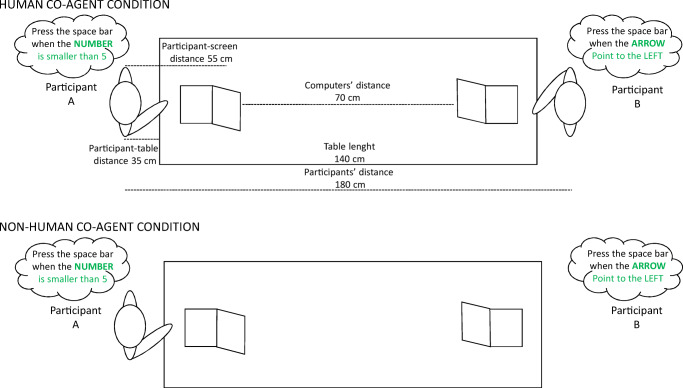
Experimental set-up for the human co-agent condition (**upper panel**) and for the non-human co-agent condition (**lower panel**)

Digits were randomly mixed with arrows in four blocks with different response rules identified above (cf. Shaki & Fischer, 2018). There were 128 trials per block, preceded by 16 practice trials. Each block contained an equal amount of numbers (56 trials: 28 go trials for Participant_A; 28 no-go trials for Participant_A) and arrows (56 trials: 28 go trials for Participant_B; 28 no-go trials for Participant_B). Block order was counterbalanced across participant. For human co-agent condition, Participant_B was a confederate (female or male, matching Participant_A’s sex), while for non-human co-agent condition, Participant_B was the computer without a second participant present (see Fig. 1).

### Procedure

For the human co-agent condition, participants sat 55 cm from their computer screen located at opposite table sides (see Fig. 1, upper panel). They were instructed to perform a go/no-go task together but with different response rules and on two different computers. At the beginning of each block they were instructed to “respond fast and accurately only in trials where a stimulus matches the current response rule.” Blocks began by displaying the same response rule on each participant screen (e.g., for Participant_A: “press space bar when digits are smaller than 5”; for Participant_B: “press space bar when arrows are facing left”). Each trial showed a randomly selected stimulus at fixation until response (go trials) or until 2,000 ms elapsed (no-go trials). Go trials for Participant_B ended after a random 400- to 600-ms interval, corresponding to pilot participants’ average response times. This was made to ensure that Participant_A could see Participant_B’s performance even though the confederate never pressed the spacebar. This latter aspect was not apparent to real Participants_A because of wearing headphones to suppress any noise and because they could not see Participant_B’s keyboard. Response speed and accuracy were recorded.

For the non-human co-agent condition, which was only performed to examine the limits of co-representing conceptual task-space, the procedure was similar, with the exception that no confederate was involved (see Fig.1, lower panel). Participants were instructed to perform a go/no-go task with the computer. Please note that in both conditions no competition between participants was assumed or implied and Participant_A was unaware that no software ran on Participant_B’s laptop.

## Analysis and results^1^

Reaction times (RTs) 2 standard deviations faster or slower than each participant’s mean (4.0% and 4.6% of all go trials for Participants_A, for the human and non-human co-agent conditions, respectively), errors (0.1% and 0.1% of all go trials for Participants_A, for human and non-human co-agent conditions, respectively; 0.8% and 0.6% of all no-go trials for Participants_A, for human and non-human co-agent conditions, respectively) and practice trials were excluded from RT analysis. Error rates were too low for analysis.

We combined response rules involving small numbers/left arrows and large numbers/right arrows into congruent conditions, and small numbers/right arrows and large numbers/left arrows into incongruent conditions.^2^

A mixed-factors ANOVA was conducted with Congruency (congruent, incongruent) and Number (large, small) as within-subject factor and Condition (human co-agent, non-human co-agent) as between-subject factor on congruency effects; we performed paired-samples t-tests to estimate congruency effects for each condition.

**Fig. 2:**
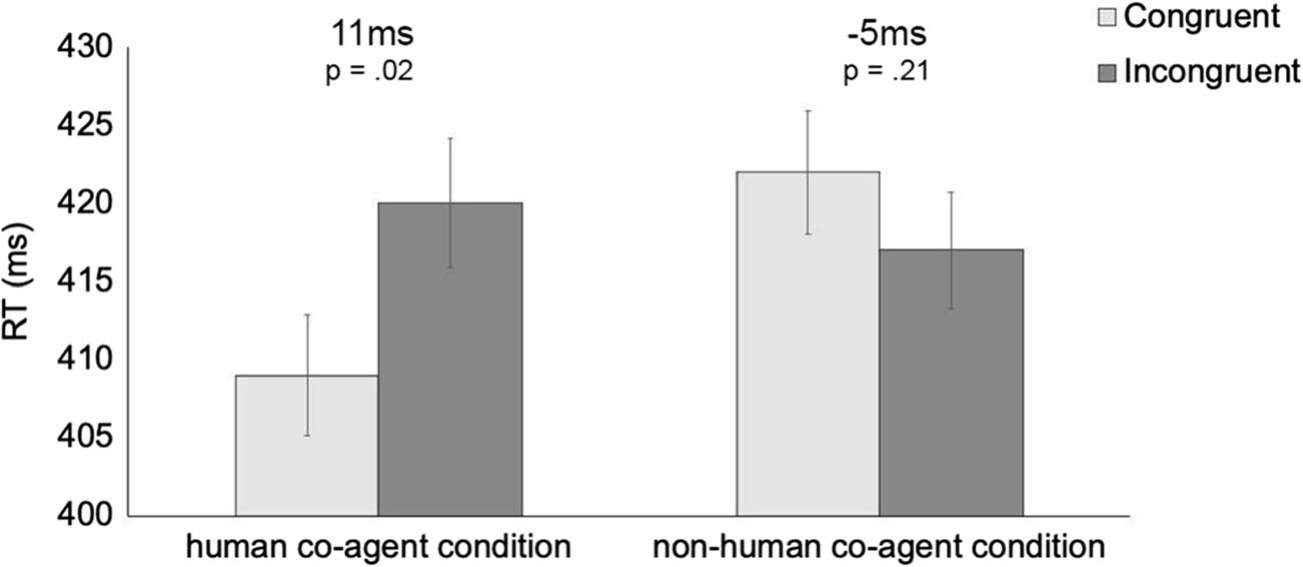
Conceptual congruency effect for human co-agent condition and non-human co-agent condition, with numerical size and associated p-value reported. Error bars indicate standard errors of the mean

The main effects of Congruency, Number, and Condition were non-significant (F-values < 1, p-values > .33), showing no difference between congruent versus incongruent trials (M = 415.49, SEM = 5.54, and M = 418.34, SEM = 5.56, respectively), with large versus small numbers (M = 418.35, SEM = 5.58, and M = 415.48, SEM = 5.55, respectively), and in human versus non-human co-agent conditions (M = 414.19, SEM = 7.57, and M = 419.64, SEM = 7.57, respectively). Interestingly, a significant interaction emerged between Congruency and Condition, F(1,46) = 7.50, *p* = .01, *η*_*p*_^*2*^ = .14, showing a larger congruency effect in human compared to non-human co-agent conditions. Specifically, human co-agents induced a reliable congruency effect of 11 ms (SEM = 4.20), t(23) = 2.57, *p* = .02, *η*_*p*_^*2*^ = .22, Cohen’s d = 0.27, showing that responses to numbers in congruent conditions were faster than responses to numbers in incongruent conditions (M = 408.75, SEM = 7.84, and M = 419.63, SEM = 7.86, respectively). Instead, non-human co-agents induced no congruency effect (-5 ms; SEM = 4.04), t(23) = -1.28, *p* = .21, *η*_*p*_^*2*^ = .066, Cohen’s d=0.13: Responses in congruent conditions did not reliably differ from those in incongruent conditions (M = 422.23, SEM = 7.84, and M = 417.06, SEM = 7.86, respectively; see Fig. 2). No other significant interactions emerged (F-values < 1, p-values > .36).

In addition, a Bayesian analysis (https://jasp-stats.org/) was conducted to quantify evidence for the alternative hypothesis (i.e., difference between congruent and incongruent conditions) relative to the null hypothesis (i.e., no difference between congruent and incongruent conditions), separately for the human co-agent (BF_10_ = 3.08) and non-human co-agent conditions (BF_10_ = .44). Thus, the alternative hypothesis is moderately (see Faulkenberry et al., 2020) more likely than the null hypothesis in the human co-agent compared to the non-human co-agent condition. While the evidence supporting the alternative hypothesis for the non-human co-agent is anecdotal (see Faulkenberry et al., 2020), here the evidence for the null hypothesis (rather than for the alternative hypothesis) is of interest. This supports the social modulation of spatial-numerical congruency effects.

## Discussion

This study built on two recent developments in search for better understanding of SNAs. First, a novel go/no-go task enabled us to study SNAs at the conceptual level and without peripheral spatial contaminations (Fischer & Shaki, 2016, 2017; Shaki & Fischer, 2018). Secondly, we utilized the insight that both space and magnitude information must be jointly activated to trigger SNAs (Pinto, Pellegrino, Marson, et al., 2019a). Here we documented that these two cognitive components can be distributed across two minds, thereby revealing novel representational constraints on SNAs.

In order to document co-representation of spatial and magnitude codes, an experiment with human and non-human co-agent conditions was implemented where magnitude processing was required by one agent and spatial processing by the co-agent. This result replicates previous work on conceptual representations of SNAs where no co-agents were involved (i.e., Fischer & Shaki, 2016, 2017; Pinto et al., 2021; Pinto, Pellegrino, Marson, et al., 2019a; Shaki & Fischer, 2018) and thereby establishes the reliability of our method. For the first time, we found the existence of *social* SNAs at the conceptual level. This differs from other joint effects, such as Simon and SNARC, which seem to originate from a shared spatial framework that emerges from overlapping feature representations of one’s own and the co-participant’s spatial responses (Sebanz et al., 2003). In that sense, previously reported joint effects were “less social” because co-participants’ actions were merely coded within shared spatial reference frames (e.g., Guagnano et al., 2010): the presence of an active confederate in peripersonal space provided a reference for coding one’s own action. In contrast, the present joint effect arose from shared *conceptual* codes after removing all sources of perceptual and motor-related congruency. Arguably, our participants represented a more abstract level of conceptual congruency between spatially directional stimuli and number magnitudes: the confederate acted outside the peripersonal space of the participant, and despite this the participant generated the representation of the other, resulting in a profoundly social effect.

Our discovery of the joint representation of SNA only occurred when both aspects of that relationship were distributed across two human minds. Thus, we found a social congruency effect based on conceptual co-representation of another participant’s rule. The social SNA emerged even when merely a single task component (magnitude) was cognitively activated, thanks to the distribution of joint conceptual congruency across two minds – although the observed effect size was smaller than in previous studies.

Our study showed a reliable difference in outcomes when comparing joint performance with a human versus a non-human co-agent. While this finding documents an interesting limitation for joint conceptual SNAs, the theoretical novelty of our study rests not in the comparison between a human and a non-human condition (or its interpretation). Instead, we show, for the first time, a joint spatial effect at the conceptual level, i.e., without any contribution whatsoever of spatial coding through lateralized referents. This is accomplished without comparison with the non-human condition. This limitation can be solved by performing, for example, an additional study in which the items are enriched.

In addition, further work is required to identify the critical ingredients, for example, whether joint conceptual spatial effects occur with avatars, robots, or other non-human agents (cf. Böffel et al., 2020; von Salm-Hoogstraeten & Müsseler, 2020) and should include manipulation checks to assess participants’ agency beliefs.

Other studies demonstrated social Simon effects depending on the degree of similarity between co-actors or their ability to reach the other participants’ responses (e.g., Hommel et al., 2009; Iani et al., in press; Müller et al., 2011; Stenzel et al., 2012; Tsai et al., 2008; Tsai & Brass, 2007). Here, shared representations emerged only when participants worked with human co-agents(for exceptions see Stenzel et al., 2012, and Müller et al., 2011), and not when they worked with computers. The absence of the congruency effect for the non-human co-agent can be interpreted as the absence of any events available for the participant to develop a conceptual congruency between numbers and space, even though both conditions shared the presence of a laptop. According to the Referential Coding Account proposed by Dolk et al. (Dolk et al., 2011; see also Dolk, Hommel, Colzato, et al., 2014b; Dolk et al., 2013) the co-actor represents a salient event that enables participants to develop a distributed spatial coding of response. Interestingly, the effect can reappear when participants merely believe they are acting with a non-humanoid co-agent such as a robot (von Salm-Hoogstraeten & Müsseler, 2020).

In conclusion, we demonstrated for the first time a shared conceptual representation that is not contaminated by peripheral spatial codes. These novel insights into the social dimension of abstract numerical thought suggest a more general consideration of social aspects of human cognition and the components necessary to activate SNAs. Future studies could assess whether processing spatially oriented objects is affected by the joint processing of numerical magnitudes across two minds, or whether individuals differ in their propensity to represent shared codes (e.g., Hartman et al., 2019).

## Footnotes

1 The data are available at: https://osf.io/3kbxz/?view_only=cc14f559d9af472f9090c2335c38205a

2 In order to examine possible order effects, we conducted an analysis taking the congruency of the first block for each participant: half of them started the experiment with a congruent response rule, and half with an incongruent response rule. A mixed-factors ANOVA was conducted with Congruency (congruent, incongruent) and Number (large, small) as within-subject factor and Condition (human co-agent, non-human co-agent) and Congruency of the First Block (congruent, incongruent) as between-subject factor. Results confirmed those reported in the article. The factor Congruency of the First Block was not statistically significant (F (1,44) = .016, *p* =.900) and neither were any interactions with this factor (F-values < 1, *p*-values > .467). We can thus assume that no order effect impacted our results.
